# Ranolazine for Prevention of Atrial Fibrillation after Cardiac Surgery: A Systematic Review

**DOI:** 10.7759/cureus.2584

**Published:** 2018-05-06

**Authors:** Nirav Patel, Jeffery Kluger

**Affiliations:** 1 Cardiology, Hartford Hospital

**Keywords:** ranolazine, post-operative cardiac surgery, atrial fibrillation, systematic review, rhythm control.

## Abstract

Postoperative atrial fibrillation (POAF) remains a major risk after cardiac surgery and is associated with an increased risk of stroke, length of stay, mortality, and cost. Ranolazine, an anti-anginal drug, also has anti-arrhythmic properties. The present study is to evaluate the effectiveness of ranolazine in preventing POAF after cardiac surgery. We searched the literature for clinical studies published up to August 2017 with a pre-defined inclusion and exclusion criteria. We identified four studies with a total of 754 patients of which 300 patients received ranolazine; there was a 14% decrease in POAF occurrence following cardiac surgery compared to 32% in the control group. Although ranolazine is an effective therapy in prevention of POAF, larger, multi-center, randomized trials are warranted.

## Introduction and background

Postoperative atrial fibrillation (POAF) is a common complication following cardiac surgery, associated with high morbidity and mortality. The development of POAF depends on the patients co-morbidities and type of surgery [1], with the highest incidence of 36%-65% in combined coronary artery bypass grafting (CABG) and valvular surgery [2-4] when compared to valvular (30%-40%) [3] or CABG only (30%) [2].

The occurrence of POAF is more frequent in patients with advanced age, left atrial enlargement, hypertension, left ventricular hypertrophy, obesity, and diabetes, and is usually evident within two to four days post surgery [5]. POAF is associated with increased risk of stroke, congestive heart failure, respiratory failure, leading prolonged hospital stay, and increase in overall health care cost [1]. Thus, chemoprophylaxis is recommended in high-risk patients [6], and therapies including beta-blockers, amiodarone, sotalol, intravenous magnesium, and steroids have proven to be effective [1,6] but they are associated with side effects without altering overall hospital stay and resource utilization [6].

Ranolazine, an anti-anginal agent, exhibits antiarrhythmic effects by inhibition of late sodium (late I_Na_) and potassium rectifier (I_Kr_) channel [7]. It produces dose-dependent block of sodium channel predominantly in the atria with significant anti-fibrillatory effect by prevention of premature atrial contraction (APC) [7]. APC is associated with atrial fibrillation (AF), and experimental and clinical studies have shown that by suppressing the APC, the incidence of atrial fibrillation (AF) is decreased [8-12]. In order to explore this hypothesis, we systematically reviewed the literature to assess the efficacy and safety of ranolazine for the prevention of POAF following cardiac surgery.

## Review

Methods

This systematic review conforms to standard guidelines and is written in accordance with the Preferred Reporting Items for Systematic Reviews (PRISMA) [13]. We performed a systematic literature search using MEDLINE, Cochrane Library (1898 to 2015), Google Scholar and ClinicalTrials.gov (1997 to 2014) by using following terms “ranolazine”, “atrial fibrillation”, “cardiac surgery”, “coronary artery bypass graft”, "valvular surgery”, and “postoperative atrial fibrillation". We reviewed all potentially relevant articles in a parallel manner by pre-defined inclusion criteria. Citations to and reference lists within the selected articles were also searched for studies that would meet inclusion criteria. All retrieved references were reviewed and evaluated the clinical outcome of interest. Pre-specified selection criteria for inclusion of studies were as follows: (1) head to-head comparisons of perioperative administration of ranolazine versus placebo or other antiarrhythmic drugs or standard therapy; and (2) study participants who underwent any cardiac surgery. Case reports, letter, experimental studies, editorials, and systematic reviews and meta-analysis were excluded. The primary endpoint was the development of POAF. Other outcomes included the incidence of adverse events and early treatment discontinuation.

Results

**Figure 1:**
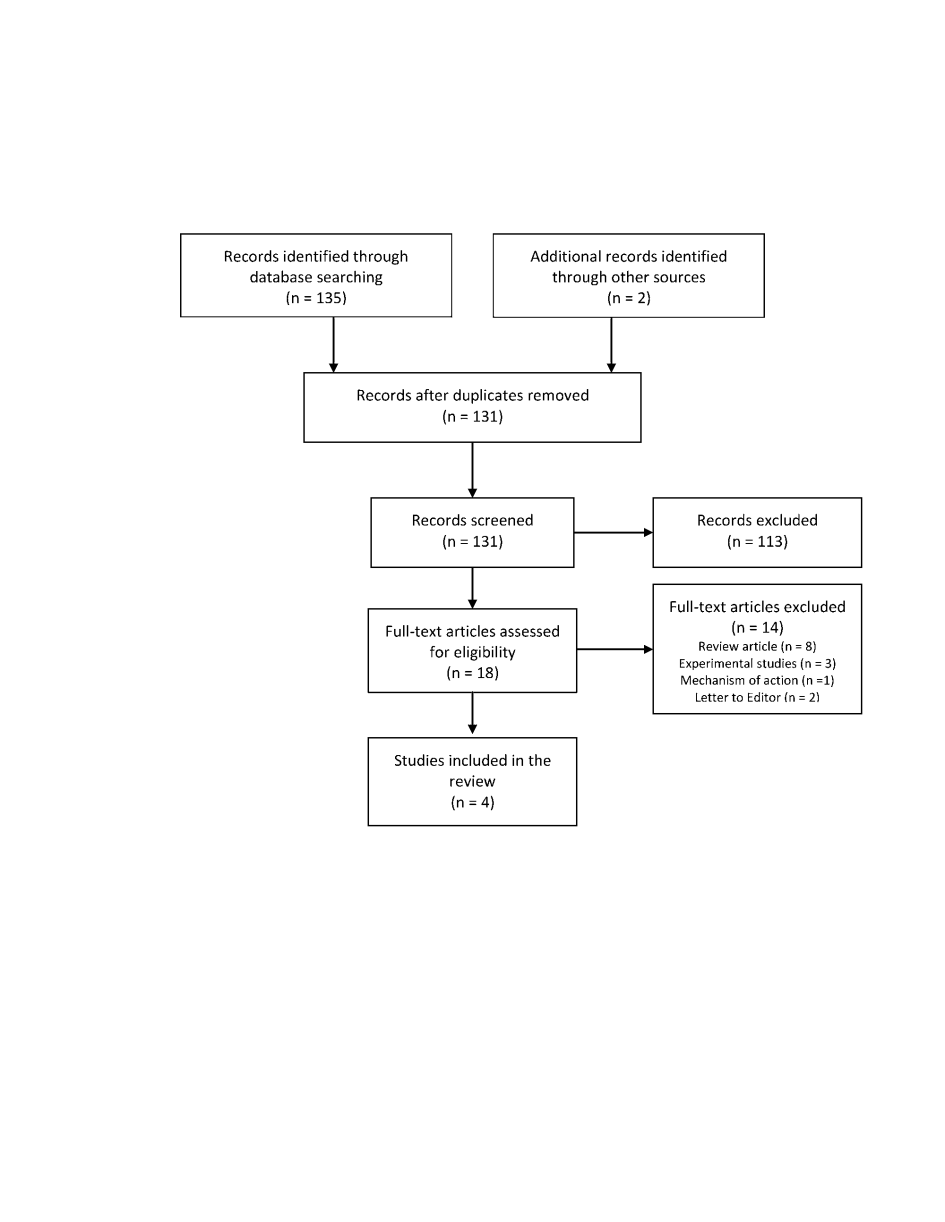
Flow chart of selected studies

A total of 131 articles were retrieved and 18 articles focused on the role of ranolazine for prevention of POAF. Exclusion of case reports, letters, experimental studies, editorials, and systematic reviews or meta-analyses and screening of titles and abstracts of these references for inclusion terms resulted in four studies (Figure 1).

The details of the clinical trials are summarized in Table 1.

**Table 1:** Details of the studies included in the systematic review AO: amiodarone; AF: atrial fibrillation; CABG: coronary artery bypass grafting; RN: ranolazine.​​​​​​​ ^€^: Surveillance was performed by continuous electrocardiography until the total length of stay. ^¥^: Surveillance was performed for at least seven days or until discharge by continuous electrocardiographic monitoring. ^£^: Duration of treatment therapy was 14 days but surveillance was conducted for 30 days. Total of 51 patients completed this cohort study.

Reference	Design	Subjects	Intervention	Duration	Type of surgery	Primary end point	Method of AF identification
Miles et al.^€^ 2011 [14]	Single-center, non-randomized retrospective cohort study	393	AO 400mg/day 7 days preoperatively and until the 10–14^th^ postoperative day. RN 1000 mg one day before or on the day of surgery and 1000 mg twice daily until the 10–14^th^ postoperative day	30 days	CABG	AF	Continuous electrocardiographic monitoring
Tagarakis et al. 2013 [15]	Prospective, single-center, single-blinded, randomized study	102	Randomized to receive either standard therapy or RN 375 mg twice daily 3 days prior to surgery until day of discharge	10 days	CABG	AF or other arrhythmias	Continuous electrocardiographic monitoring
Hammond et al.^¥^ 2015 [16]	Single-center, retrospective cohort study	205	RN group received RN 1000 mg on the morning of surgery and 1000 mg twice daily until the 7^th^ postoperative day. Non-RN group received standard therapy	7 days	CABG, valve and combination surgeries	AF	Continuous electrocardiographic monitoring
Bekeith et al.^ £^ 2015 [17]	Single-center, double-blinded, randomized trial	54	Randomized to receive either placebo or RN 1000 mg twice daily 48 hours prior to surgery to 14th postoperative day	14 days	CABG and/or aortic valve replacement surgeries	AF	Holter monitoring

Baseline characteristics of the patients in the clinical trials are presented in Table 2.

**Table 2:** Baseline characteristics of patients AF: atrial fibrillation; CABG: coronary artery bypass grafting; LVEF: left ventricular ejection fraction; NR: not reported; RN: ranolazine. Values are reported as mean ±SD or n (%). ^£ ^: The data above is a representation of both the RN and control group combined.

		Age, years	Male (%)	Hypertension (%)	Diabetes (%)	History of AF in %	LVEF (%)
Miles et al. 2011 [14]	RN (n=182)	66.7±9.3	127 (70)	158 (87)	71 (39)	4.5	57.7±9.8
	Control (n=211)	64.9±10.9	162 (77)	182 (86)	76 (36)	7.6	54.7±12.7
Tagarakis et al. 2013 [15]	RN (n=34)	69±7	24 (71)	NR	NR	NR	52.6±8.6
	Control (n=68)	67±8	45 (66)	NR	NR	NR	53.8±9.4
Hammond et al. 2015 [16]	RN (n=57)	60.3±11.1	38 (67)	45 (79)	20 (35)	1.8	NR
	Control (n=57)	59.6±11.5	38 (67)	48 (84)	20 (35)	1.8	NR
Bekeith et al.^ £^ 2015 [17]	RN (n=27)	64.3±11.4	44 (81)	48 (89)	22 (41)	NR	46.4±14.6
	Control (n=27)						

All studies were single-centered, one prospective [15], two retrospective [14,16] and included one randomized trial [17]. The total sample size was 754 ranging from 54 to 393 with the duration of follow up from seven to 30 days (median: 17 days; interquartile range (IQR): 18.25). The mean age was 65.4 years (IQR: 6.5) and was predominantly male (81%). The mean LVEF was 53.04% (IQR: 6.5) and left atrial diameter 34.9 millimeters (mm) in the experiment as compared to 33.8 mm in the control group [15]. The co-morbidities were indifferent between the two groups with hypertension being more prevalent at 86% and diabetes at 37%. Two studies included patients with a history of AF [14,16] with a total of 10% in the control froup and 7% in the treatment group. The control group treatment varied among the studies. Standard therapy was used in two studies [15,16] the results of which were not disclosed; in one study the comparison was against amiodarone [17] and the other was against placebo [17]. Ranolazine dosing, initiation, and termination varied among the studies as follows: (1) ranolazine 1000 mg a day before surgery or on the day of surgery and then increased to 1000 mg twice daily for 10 to 14 days [14]; (2) ranolazine 1000 mg on the morning of the surgery and then twice daily for seven days [16]; (3) ranolazine 100 mg twice daily 48 hours prior to surgery for 14 days [17]; (4) ranolazine 375 mg twice daily, 72 hours prior to surgery, until the day of discharge for 10 days [15]. CABG was more common in the control group at 53% (369/700) as compared to the treatment group of 37% (259/700), the valvular surgery in the treatment and control group was 3% versus 5%, respectively [14-16]. Combined CABG and valvular surgery were indifferent in both groups [14-16].

On an average, the incidence of POAF was lower in the ranolazine group by 14% when compared to 32% in the non-ranolazine group. Hypotension was noted in the treatment group but resolved within 72 hours [16] with no other adverse effect noted and no difference in overall mortality and 30-day readmissions.

Discussion

POAF is typically a transient, reversible phenomenon that may develop in patients at risk. Multiple mechanisms are involved in the development of POAF including pericardial inflammation, catecholamine surge, autonomic imbalance, and interstitial mobilization of fluid with resultant changes in volume [5]. POAF is usually asymptomatic but is associated with increased morbidity and mortality [1]. Although perioperative beta-blockers, amiodarone, sotalol, calcium channel blockers, and magnesium have been used for the prevention of POAF, they are limited by adverse events (including hypotension and bradycardia) [1,5] and by their suboptimal efficacy and potential for pro-arrhythmia risk [7].

Ranolazine, a piperazine derivative, exerts antiarrhythmic effects in both the ventricles and the atria and has been evaluated to prevent POAF. Although the exact mechanism for anti-arrhythmic effect is unknown, in the atrial tissue, ranolazine may suppress AF primarily by inhibiting peak sodium current (INa) and also causes inhibition of the delayed rectifier potassium (Ikr) current, leading to a decrease in atrial myocardium sensitivity and vulnerability to AF. Ranolazine reduces acetylcholine (Ach) mediated AF through different mechanisms by inhibiting INa, Ikr and, peak INa leading to a reduction of action potential upstroke and an increase in the diastolic threshold of excitation and post-repolarization refractoriness [18].

Several studies have demonstrated the clinical efficacy of ranolazine for the prevention of AF and was associated with a significant reduction of supraventricular tachyarrhythmia (p < 0.001) as well as a 30% reduction in new-onset AF (p = 0.08) in the Metabolic Efficiency with Ranolazine for Less Ischemia in Non-ST-elevation acute coronary syndromes – Thrombolysis in Myocardial Infarction (MERLIN-TIMI) 36 trial [10]. Murdock et al. demonstrated that ranolazine is helpful in maintaining sinus rhythm in patients with resistant AF [11]. Another study by the same authors suggested that a single dose of ranolazine 2000 mg is effective in converting 77% of AF patients to sinus rhythm with no significant adverse drug reactions [12]. The Ranolazine in AF Following An ELectricaL CardiOversion (RAFFAELLO) study assessed the safety and efficacy of ranolazine in the prevention of AF recurrence after successful electrical cardioversion [19]. AF recurred in 56.4%, 56.9%, 41.7%, and 39.7% of patients in the placebo, ranolazine 375 mg, ranolazine 500 mg, and ranolazine 750 mg groups, respectively [19]. The reduction in overall AF recurrence in the combined 500-mg and 750-mg groups was of non-significance compared to the placebo group (P = 0.053) and significant compared to 375-mg group (P = 0.035) [15].

The results from this systematic review must be evaluated within the context of its potential limitations. Firstly, the included studies were single-sited, retrospective observational, and prospective evaluations of the report of POAF after ranolazine therapy. These studies included younger patients with a mean age of less than 70 years and mean left ventricular ejection fraction >50% suggesting these patients were at low risk for developing POAF. Other important characteristics of left atrial size were not included. Many of the studies had small sample sizes with varied duration times and surveillance methods. Therapy in control group is diverse among all the studies and two studies [15-16] did not disclose the type of “standard therapy” being implemented. The dosing, initiation and termination of ranolazine also varied among the studies.

## Conclusions

Although limited by lack of size and adequate control groups, the literature does suggest that ranolazine may be an efficacious and safe therapy for the prevention of POAF following cardiac surgery. However, multi-center, large, randomized controlled trials are warranted.
